# Unusual accumulation of a wide array of antimicrobial resistance mechanisms in a patient with cytomegalovirus-associated hemophagocytic lymphohistiocytosis: a case report

**DOI:** 10.1186/s12879-020-04966-z

**Published:** 2020-03-20

**Authors:** Mohammad Rubayet Hasan, Manu Somasundaram Sundaram, Sathyavathi Sundararaju, Kin-Ming Tsui, Mohammed Yousuf Karim, Diane Roscoe, Omar Imam, Mohammad A. Janahi, Eva Thomas, Simon Dobson, Rusung Tan, Patrick Tang, Andres Perez Lopez

**Affiliations:** 1 Department of Pathology, Sidra Medicine, Level 2M, Office H2M-24093, PO BOX 26999, Doha, Qatar; 2 Weill Cornell Medical College in Qatar, Doha, Qatar

**Keywords:** Multidrug-resistant organism, Antiviral resistance, Cytomegalovirus, Hemophagocytic lymphohistiocytosis

## Abstract

**Background:**

Infections with multidrug-resistant organisms (MDRO) pose a serious threat to patients with dysregulated immunity such as in hemophagocytic lymphohistiocytosis (HLH), but such infections have rarely been comprehensively characterized. Here, we present a fatal case of HLH secondary to cytomegalovirus (CMV) infection complicated by both anti-viral drug resistance and sepsis from multiple MDROs including pandrug-resistant superbug bacteria.

**Case presentation:**

A previously healthy, six-year-old boy presented with a 45-day history of fever prior to a diagnosis of hemophagocytic lymphohistiocytosis and hemorrhagic colitis, both associated with CMV. On hospital admission, the patient was found to be colonized with multiple, multidrug-resistant (MDR) bacteria including vancomycin-resistant enterococci (VRE) and carbapenamase-producing organisms (CPO). He eventually developed respiratory, urine and bloodstream infections with highly drug-resistant, including pandrug-resistant bacteria, which could not be controlled by antibiotic treatment. Antiviral therapy also failed to contain his CMV infection and the patient succumbed to overwhelming bacterial and viral infection. Whole genome sequencing (WGS) of the MDR bacteria and metagenomic analysis of his blood sample revealed an unusual accumulation of a wide range of antimicrobial resistance mechanisms in a single patient, including antiviral resistance to ganciclovir, and resistance mechanisms to all currently available antibiotics.

**Conclusions:**

The case highlights both the risk of acquiring MDR superbugs and the severity of these infections in HLH patients.

## Background

Antimicrobial resistance (AMR) is one of the most important public health challenges of current times as the burden of infectious diseases with multidrug-resistant organisms (MDRO) is increasing at an alarming rate. Globally, approximately 500,000 people die each year due to drug-resistant infections and, if not controlled, these deaths are predicted to exceed 10 million by 2050 [1]. Particularly vulnerable are patients with immune deficiency or dysregulation whose inability to fight infections leads to increased risk of disseminated infection and greater dependency on antimicrobial therapy [2]. Hemophagocytic lymphohistiocytosis (HLH) is a potentially life-threatening condition characterized by overactivation of lymphocytes and macrophages that results in dysregulation of inflammatory responses [3]. While these factors likely put HLH patients at high risk of infection with MDROs, no such reports has been published to date and the clinical course of such infections in these patients remains unknown. Here we report severe infections with multiple MDROs in a patient with cytomegalovirus (CMV)-associated HLH. We also show an unusual accumulation of a wide-array of antiviral and antibiotic resistance mechanisms in a single patient based on data generated by shotgun metagenomic sequencing of blood from the patient and whole genome sequencing (WGS) of MDROs isolated from the patient.

## Case presentations

A previously healthy 6-year-old boy presented at our hospital in August 2018 with a 45-day history of intermittent, high-grade fever without a clear source, accompanied by loss of appetite, weight loss, painful tongue ulcers, diffuse abdominal pain and intermittent left calf muscle pain. He was a resident of Qatar who had just returned from a family holiday in India, where he was hospitalized twice due to fever of unknown origin (FUO) and received intravenous (IV) ceftriaxone and amikacin without any improvement. On examination, he looked moderately ill and pale, and was found to have mouth ulcers and splenomegaly. Initial laboratory investigations revealed neutropenia, thrombocytopenia, normocytic normochromic anemia with high ferritin, elevated liver enzymes and C-reactive protein (CRP).

**Fig. 1:**
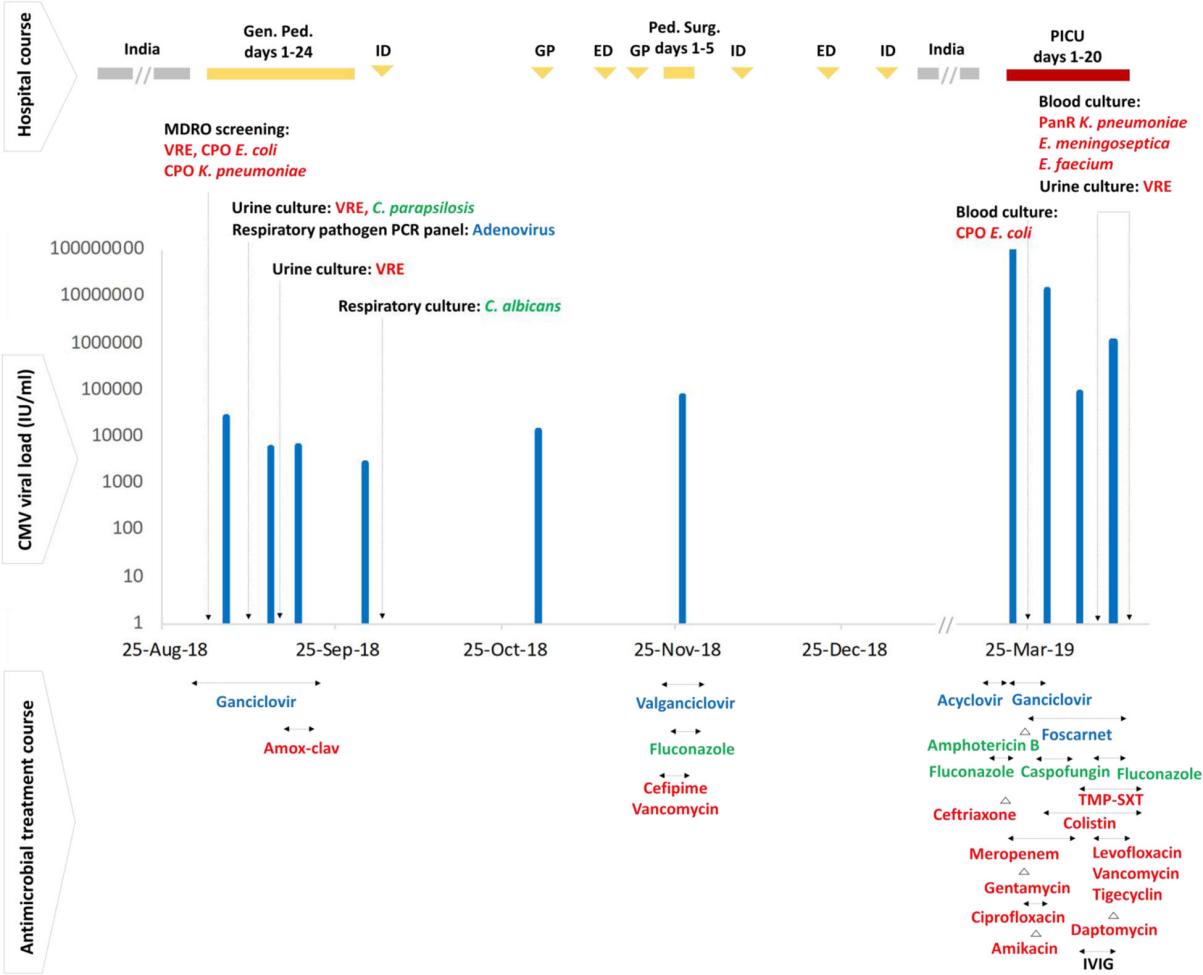
Hospital course, microbiology and antimicrobial treatment history. ID, infectious disease clinic; ED, emergency department, GP, general pediatrics clinic; Gen. Ped., general pediatrics unit; Ped. Surg., pediatric surgery unit; PICU, pediatric intensive care unit. Orange bars, orange arrows and red bar show inpatient days, outpatient visits and PICU days, respectively. Grey bars show approximate timing of hospitalization in India

On admission, surveillance cultures for MDROs were positive for vancomycin-resistant *Enterococcus faecium* (VRE) and carbapenemase-producing *Escherichia coli* (NDM1) and *Klebsiella pneumoniae* (OXA-48) (Fig. 1). He was also found to be positive for cytomegalovirus (CMV) IgM with a viral load of 27,946 IU/mL. Bone marrow examination performed twice showed hypocellularity with myeloid preponderance and no morphologic evidence of malignancy. Immunological workup revealed severe reduction in CD19+ B-cells and CD16 + CD56+ NK cells. Genetic testing (Invitae, San Franscico, USA) with a primary immunodeficiency (PID) panel comprised of 207 genes revealed three variants of uncertain significance. His liver enzymes were elevated. Liver needle biopsy and electron microscopy revealed mild steatosis with steatohepatitis. Patchy sinusoidal dilation with hepatocellular plate atrophy was noted. Wilson’s disease was excluded by genetic testing. All bacterial, mycobacterial and fungal cultures of bone marrow were negative. Extensive infectious disease workup during the course of his hospitalization were negative except that the respiratory pathogen PCR panel was positive for adenovirus and rhinovirus and that VRE and *Candida* spp. were isolated from his urine culture. Antiviral therapy for CMV viremia was started with IV ganciclovir for 3 weeks. CMV viral load dropped to 6167 IU/ml. Patient showed symptomatic improvement and was discharged with oral valganciclovir for another 3 weeks.

Four months post discharge, he developed fever and a productive cough. He had several visits to the emergency department, and infectious disease and hematology outpatient clinics, and was briefly hospitalized again for fever, recurrent respiratory and *Candida* infections, recurrent thrombocytopenia and suspected immunodeficiency. After discharge, he continued to have intermittent fever, and his parents took him to India for further evaluation. He was diagnosed with hemophagocytic lymphohistiocytosis (HLH) secondary to CMV infection, and started treatment with dexamethasone and cyclosporine in February 2019. In order to manage the CMV viremia, he was also treated with multiple antivirals including cidofovir and foscarnet.

The child was re-admitted to Sidra Medicine after 1 month of therapy for HLH with a one-week history of fever, hematochezia and fatigue. On the same day, he developed massive rectal bleeding with blood clots and required resuscitation and was transferred to the pediatric intensive care unit (PICU). The rectal bleeding and hemodynamic instability required blood and platelet transfusions and urgent colonscopy, which showed erosion and signs of colitis. CMV-induced hemorrhagic colitis was suspected, with the possibility of transmural infiltration of the bowel with hemophagocytic cells. He was intubated on day two. On PICU day three, the patient became hypotensive during interventional radiology and required resuscitation. Blood culture was positive with multidrug-resistant *E. coli*. Prolonged antiviral therapy and the lack of response led to a suspicion of CMV ganciclovir resistance, so foscarnet was started.

He was extubated 5 days after intubation but on PICU day 16, he was reintubated after developing pulmonary hemorrhage. The chest x-ray showed worsening pulmonary hemorrhage and he developed catecholamine-resistant shock that required initation of steroids. Also, he continued to have bleeding per rectum requiring continuous support with blood products. A CT angiogram showed bleeding from the terminal ileum and colitis in the large intestine. He was put on total parenteral nutrition. Surgical intervention for the gastrointestinal bleeding was considered too risky. Thus, he was conservatively managed with octreotide and esomeprazole infusion and blood product transfusions. He also developed acute kidney injury likely from the combination of nephrotoxic medications, hematuria and infection, and continued to be anuric, requiring continuous renal replacement therapy. He also developed microangiopathic hemolytic anemia requiring 5 cycles of plasmapheresis. For HLH, his treatment was escalated to increasing doses of dexamethasone, intravenous immunoglobulin (IVIG) and cyclosporine.

**Fig. 2:**
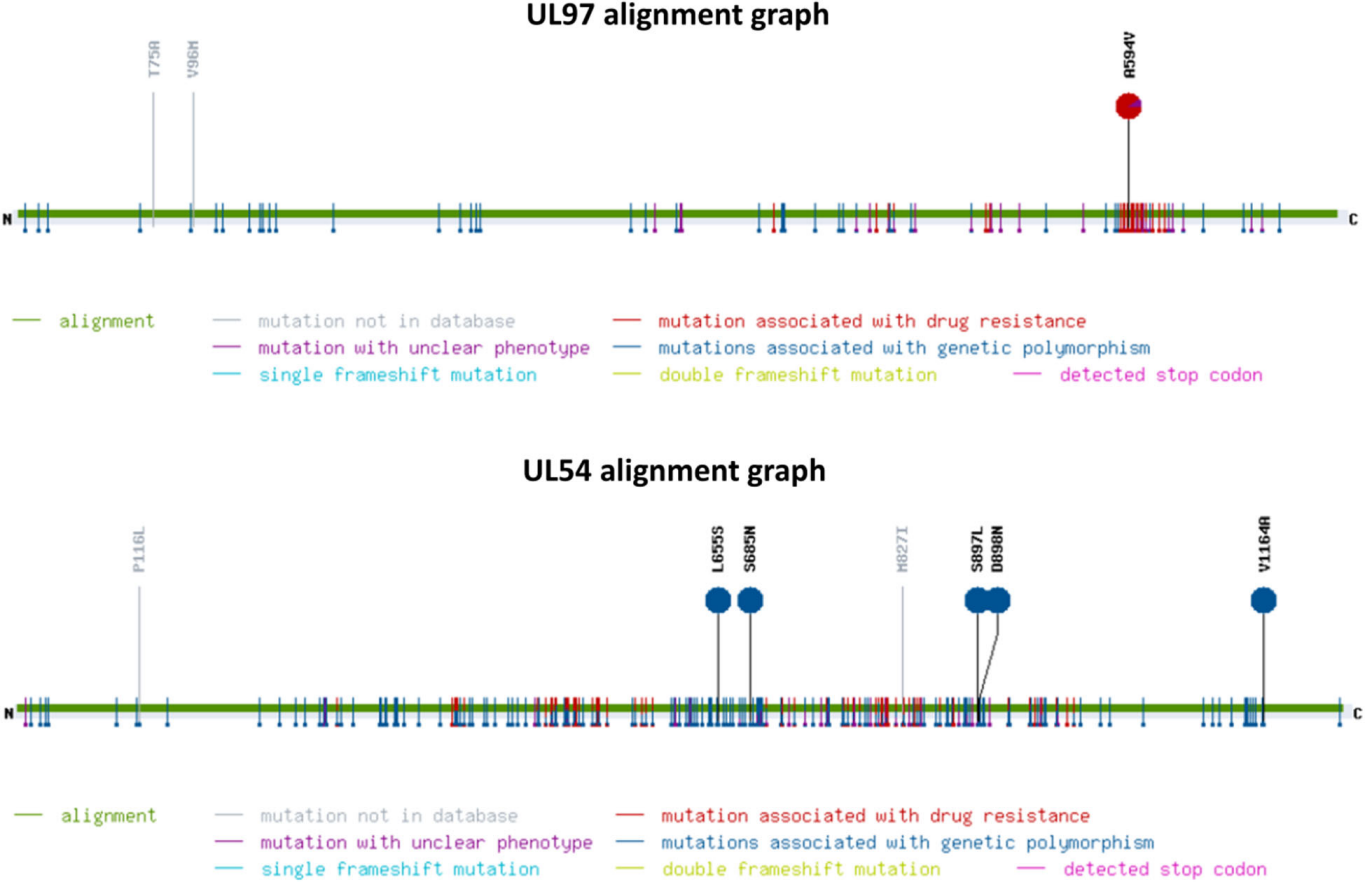
Genotype and antiviral resistance profiles of the cytomegalovirus strain. Nucleic acid extract from patient serum was subjected to NGS library preparation using Nextera XT kit (Illumina, USA) and sequencing was performed on a MiSeq (Illumina). Paired sequence reads were mapped to UL97 and UL54 sequences (Gene ID 3077517 and GenBank accession ABV71585.1, respectively) to obtain corresponding gene sequences from the patient’s CMV strain. The sequences were then analyzed by using an online mutational resistance analyzer (MRA) available from the University of Ulm, https://www.informatik.uni-ulm.de/ni/mitarbeiter/HKestler/mra/app/index.php?plugin=form [5]. Ganciclovir resistance was confirmed by the presence of the A594V mutation in UL97 [6]

Microbiological findings during this time were remarkable for multiple cultures positive for MDROs. The first was a positive blood culture on PICU day 5 with an extensively drug resistant, carbapenemase-producing *E. coli,* which prompted the addition of colistin to his antimicrobial treatment (Fig. 1). This was followed by multiple positive blood cultures with *Elizabethkingia meningoseptica*, *E. faecium* and pandrug-resistant *K. pneumoniae,* and a positive urine culture with VRE*.* Whole genome sequencing (WGS) was performed on several multi-dug resistant bacteria isolated from the patient at various time points showing accumulation of resistance mechanisms to almost all classes of antibiotics used to treat these bacteria (Table 1, Supplemental methods). Shotgun metagenomic sequencing performed on serum on day 6, revealed an A594V mutation in the UL97 gene of CMV (Fig. 2, Supplemental methods), which is known to confer resistance to ganciclovir. As a result, ganciclovir was discontinued leaving foscarnet as his anti-viral treatment. IV levofloxacin and vancomycin was added to cover *E. meningoseptica* and VRE, along with prophylactic trimethoprim/sulfamethoxazole and antifungal treatment. His CMV titres continued to be high in spite of antiviral therapy. Over the course of the last few days of PICU, the patient continued to deteriorate clinically: pulmonary bleeding persisted with intermittent respiratory acidosis despite maximal ventilator support. He continued to be hypotensive despite inotropic support, remained anuric and continued to have gastrointestinal (GI) bleeds. The patient passed away on PICU day 20 from multiorgan failure associated with sepsis with highly drug-resistant bacteria.

**Table 1:** Genetic mechanisms of antibiotic resistance in bacteria isolated from the patient throughout hospital course

Specimen/Organism/Date of collection	Antibiotic resistance phenotype by AST	Genotype/Resistance						
		Sequence type	Β-lactam antibiotics	Aminoglycosides	Quinolones	Macrolides	Glycopeptides	Other drugs
MDRO screen ***Enterococcus faecium*** 29/08/2018	AMP, LZD, GENS, VAN	C16; *E. faecium* ST unknown		aac(6′)-aph(2″) ant(6)-Ia	parC 80I gyrA 83Y	Erm (B), erm (T)	VanHax	aph(3′)-III Tet(L)
MDRO screen ***Escherichia coli*** 29/08/2018	AMP, AMC, TZP, AZT, CXM, FEP, FOX, CAZ, CRO, CIP, LVX, GEN, ETP, IPM, MEM, SXT	CP18; *E coli* ST410	CMY-2 CTX-M-15 NDM-5 OXA-1 TEM-1B	aac(3)-IId, aac(6′)-Ib-cr aadA5, aph(3″)-Ib aph(6)-Id	aac(6′)-Ib-cr gyrA 83 L gyrA 87 N parC 80I			dfrA17 sul1, sul2
MDRO screen ***Klebsiella pneumoniae*** 29/08/2018	AMK, AMP, AMC, TZP, AZT, CXM, FEP, FOX, CAZ, CRO, CIP, LVX, GEN, ETP, IPM, MEM, SXT	CP19; *K. pneumoniae* ST2096	CTX-M-15 OXA-1 OXA-232 SHV-28 TEM-1A	aac(6′)-Ib-cr aadA2 armA	aac(6′)-Ib-cr oqxAB gyrA 83Y gyrA 87G parC 80I			dfrA1, dfrA12, dfrA14 sul1 tet(L)
Urine ***Enterococcus faecium*** 09/09/2018	AMP, LZD, GENS, VAN, CIP, NIT, TET	C17; *E. faeceum* ST80		aac(6′)-aph(2″) aph(3′)-III	gyrA 83I parC 80I	erm(A), erm(B), erm(T)	VanHAX	tet(L)
Blood (PICC line) ***Escherichia coli*** 26/03/2019	AMK, AMP, AMC, TZP, AZT, CFZ, CFL, CXM, FEP, FOX, CAZ, CRO, CIP, LVX, GEN, ETP, IPM, MEM, SXT, CZA, C/T, FOF	CP48; *E. coli* ST405	blaNDM-5 blaTEM-1B	aadA2	gyrA 83 L gyrA 87 N parC 80I	erm(B)		dfrA12 sul1
Blood (peripheral) ***Klebsiella pneumonaie*** 06/04/2019	AMK, AMP, AMC, TZP, AZT, CXM, FEP, FOX, CAZ, CRO, CIP, LVX, GEN, ETP, IPM, MEM, SXT, FOF, TGC, CST	C12; *K pneumoniae* ST395	CTX-M-15 NDM-5 OXA-1 OXA-232 SHV-11 TEM-1B	aac(6′)-Ib-cr aadA2 aph(6)-Id rmtB strA	aac(6′)-Ib-cr oqxAB gyrA 83I parC 80I	erm(B)		dfrA12 dfrA7 sul1
Blood (port a cath) ***Elizabethkingia meningoseptica*** 06/04/2019	AMK, AZT, FEP, CAZ, CRO, GEN, IPM, MEM	C11R; *E. meningoseptica*	B-14 GOB-1					CME-2

*AMK* amikacin, *AMC* amoxicillin-clavulanic acid, *AMP* ampicillin, *SAM* ampicillin-sulbactam, *AZM* azithromycin, *ATM* aztreonam, *CFZ* cefazolin, *CFL* cephalexin, *FEP* cefepime, *CTX* cefotaxime, *FOX* cefoxitin, *CAZ* ceftazidime, *CZA* ceftazidime-avibactam, *C/T* ceftolozane-tazobactam, *CRO* ceftriaxone, *CXM* cefuroxime, *LEX* cephalexin, *CEF* cephalothin, *CHL* chloramphenicol, *CIP* ciprofloxacin, *CLR* clarithromycin, *CLI* clindamycin, *CST* colistin, *DOX* doxycycline, *ETP* ertapenem, *ERY* erythromycin, *FOF* fosfomycin, *GEN* gentamicin, *GENS* gentamicin synergy, *IPM* imipenem, *KAN* kanamycin, *LVX* levofloxacin, *LZD* linezolid, *MEM* meropenem, *NIT* nitrofurantoin, *OXA* oxacillin, *PEN* penicillin, *PIP* piperacillin, *TZP* piperacillin-tazobactam, *STR* streptomycin, *TET* tetracycline, *TGC* tigecycline, *TMP* trimethoprim, *SXT* trimethoprim-sulfamethoxazole, *VAN* vancomycin

*Bacterial whole genome sequencing (WGS) was performed as described in the supplemental methods and data were analyzed as described previously [4]

## Discussion and conclusions

HLH is characterized by dysregulation of the immune system whereby hematopoietic cells are phagocytosed by activated macrophages and lymphocytes [7]. Primary or familial HLH may be inherited in an autosomal recessive manner and are grouped into 5 types based on the affected gene. Type 1 HLH is caused by a defect in chromosome 9, while types 2, 3, 4 and 5 are known to be caused by mutations in familial HLH (fHLH) genes *PRF1, UNC13D, STX11 and STXBP2* genes, respectively [7]. At the initial presentation to our hospital in June 18, 2018 with CMV viremia, the patient did not meet criteria for HLH. This diagnosis was made when abroad in India, and treatment started there. Subsequent review identified that the patient developed 8/8 HLH-2004 criteria [8]. In our patient, the history of recurrent infection, the CMV viremia, the clinical diagnosis of HLH and marked reduction in B-cell and NK-cell numbers could suggest an underlying primary immunodeficiency (PID) disorder. However, a sample sent to a referral laboratory to test for PID genes including above HLH-associated genes did not identify significant mutations in known PID genes, suggesting that HLH in our patient was CMV-associated. HLH in an immunocompetent patient secondary to CMV infection is extremely rare but has been reported in the literature [9].

What was unique in our patient compared to other reported CMV-associated HLH cases was the overwhelming infection with MDROs. On hospital admission, the patient was found to be colonized with multiple MDROs including VRE, and carbapenamase-producing *Enterobacteriaceae*, which may have been acquired during his previous hospital course in India. WGS revealed that the VRE isolate harbored the vanHAX gene cluster that encodes VanA, providing high level resistance to vancomycin and teicoplanin. Apart from vancomycin resistance, the isolate also possessed genes encoding resistance to macrolides, lincosamides, tetracyclines and aminoglycosides (Table 1). Phenotypically, the isolate was also resistant to linezolid but we were unable to identify the genetic determinant. The *E. coli* and *K. pneumoniae* isolates harbored genes encoding a wide array of antimicrobial resistance mechanisms affecting the vast majority of antibiotic classes, including CTX-M-15 and NDM β-lactamases, the most common extended-spectrum β-lactamases and carbapenemases, respectively, found in *Enterobacterales* in India [10, 11]. Notably, the *E. coli* isolate had 5 different modifying enzymes conferring resistance to aminoglycosides, which has rarely been reported [12]. There were also several narrow host range plasmids belonging to the IncF family (replicons FIA, FIB and FII) in the *E. coli* and *K. pneumoniae* strains (data not shown) [10]. Since these plasmids can simultaneously harbor most of the genes detected and can be transferred both within the same species and between both species, it is presumed that they played a role in the acquisition and subsequent exchange of resistance determinants among these isolates.

Consequently, our patient developed respiratory, urine and bloodstream infections with highly drug-resistant, including pandrug-resistant, bacteria, which could not be controlled by antibiotic treatment. Although the patient initially responded to the antiviral drug ganciclovir as reflected by a drop in CMV viral load (Fig. 1), he later became non-responsive to antiviral treatment. Due to the severity of the CMV infection despite ganciclovir treatment, real-time metagenomic analysis was performed, revealing ganciclovir resistance and allowing for tailoring of the antiviral therapy. Although the UL97 mutation detected in this case is well described in the literarture for its association with ganciclovir resistance, the reduced absorption of the drug because of CMV-assiciated enterocolitis may have contributed to the development of resistance as well [13].

By WGS, the VRE isolate from his urine and the *E. coli* and *K. pneumoniae* isolates from his blood culture were found to be of different sequence types compared to the isolates the patient was previously found to be colonized with, but the later clinical isolates harbored both the resistance mechanisms found in the colonizing strains as well as newly acquired resistance mechanisms. In particular, the pandrug-resistant *K. pneumoniae* isolated from the patient’s blood had NDM β-lactamases in addition to OXA-48 β-lactamases. Phenotypically, the isolate was resistant to all currently used antibiotics including the last resort antibiotic, colistin. We were unable to detect the *mcr* gene that is commonly known to encode for colistin resistance and the genetic determinants of colistin resistance remained unknown in this study. While the exchange of resistance determinants via horizontal gene transfer is a possibility, the invasive superbug infections in our patient may have been facilitated by his travel history as well as his CMV-associated enteropathy, which eventually culminated with untreatable sepsis, multiorgan failure and the untimely death.

Our study has a few limitations. Although the patient was tested for a panel of genes for PID, WGS was not performed to look for other genetic causes of HLH or PID, outside of the commonly known mutations. Also, the clinical course of our patient in India was not well documented and details regarding antibiotic and antiviral treatment of the patient are unknown. However, it appears most likely that the patient acquired MDROs while seeking medical treatment in India, where MDRO infection is frequently associated with hospitalization [14–16]. Therefore, the risks of MDRO infections associated with medical tourism to regions with high rates of antibiotic resistance should be discussed with patients, who are at higher risk for complications from these organisms. It is important that the travel and other relevant history be elicited from patients so that appropriate screening measures for MDROs can be implemented. Strict infection control measures are necessary to reduce nosocomial transmission of MDROs, especially in centers caring for immunocompromised or critically ill patients. In our institution, we perform risk-based screening for MDROs, implement appropriate isolation measures to prevent spread to other patients and health care workers, and perform WGS to monitor for nosocomial transmission. This case depicts the dire circumstances associated with severe infection with MDR superbugs in a particularly vulnerable patient, and underscores the need for urgent measures to prevent the development of antibiotic resistnace through appropriate use of antimicrobials and to prevent the spread of MDROs through surveillance and implementation of appropriate infection control measures.

## Supplementary information

**Additional file 1.** Supplemental methods.

## Data Availability

Bacterial whole genome sequence (WGS) data and metagenomics data are available under Bioprojects PRJNA564977 and PRJNA576033 in DDBJ/ENA/GenBank with the accession numbers SRX6852761, SRX6852760, SRX6852759, SRX6852758, SRX6852757, SRX6852756, SRX6852755 and SRR10236626, respectively.

## Abbreviations

AMR: Antimicrobial resistance
CMV: Cytomegalovirus
CPO: Carbapenamase-producing organism
CRP: C- reactive protein
CT: Computed tomography
FUO: Fever of unknown origin
GI: Gastrointestinal
HLH: Hemophagocytic lymphohistiocytosis
IV: Intravenous
IVIG: Intravenous immunoglobulin
MDR: Multi-drug resistant
MDRO: Multi-drug resistant organism
NK: Natural killer
PCR: Polymerase chain reaction
PICU: Pediatric intensive care unit
PID: Primary immunodeficiency
VRE: Vancomycin-resistant *Enterococcus*
WGS: Whole genome sequencing
