# Supplementary material for: Unusual accumulation of a wide array of antimicrobial resistance mechanisms in a patient with cytomegalovirus-associated hemophagocytic lymphohistiocytosis: a case report
Source: BMC Infect Dis. 2020 Mar 20;20:237. doi: 10.1186/s12879-020-04966-z (PMC7083060; doi:10.1186/s12879-020-04966-z)
Supplement: Supplementary file 1 — Additional file 1. Supplemental methods. [file 12879_2020_4966_MOESM1_ESM.docx]

**Supplemental Methods:**

Written informed consent was obtained from the parents of the patient prior to the review of patient’s charts and case presentation. Metagenomic sequencing analysis of clinical sample and whole genome analysis of bacteria isolated from the patient were approved by the institutional review board (IRB) of Sidra Medicine, as part of other ongoing research projects. Unless otherwise mentioned, all laboratory tests were performed according to established procedures in the Pathology Department of Sidra Medicine, or its partner referral centers. Bacteria were isolated in sheep blood agar medium (BD, USA), and DNA from bacteria and a serum specimen were extracted using the automated nucleic acid extraction platform NucliSENS easyMag (bioMérieux) and DNA concentration was quantified using a Qubit fluorometer (Thermo Fisher, Waltham, MA). The paired-end DNA libraries were constructed from bacterial DNA or serum DNA extracts using the Nextera XT kit (Illumina, San Diego, CA) according to the manufacturer’s instructions and sequenced on an Illumina MiSeq machine with 2 × 300-bp cycles and 2 × 250-bp cycles, respectively. For the metagenomics sample, prepared NGS library was quantified by Kappa qPCR according to manufacturer’s instructions (Roche). NGS libraries were normalized according to manufacturer’s instructions. Metagenomic sequence data was analyzed using Geneious 11.1.5. software (Geneious) to obtain UL97 and UL54 sequences in the CMV strain in the patients’ blood. The UL97 and UL54 sequences retrieved from metagenomic sequences were uploaded to <https://www.informatik.uni-ulm.de/ni/mitarbeiter/HKestler/mra/app/index.php?plugin=form> for mutation analysis and drug resistance prediction [9,10]. Bacterial whole genome sequencing data were analyzed as described previously [11].
